# Research on Emergency Response Policy for Public Health Emergencies in China—Based on Content Analysis of Policy Text and PMC-Index Model

**DOI:** 10.3390/ijerph191912909

**Published:** 2022-10-09

**Authors:** Ying Zhao, Lin Wu

**Affiliations:** School of Sociology, Wuhan University, Wuhan 430072, China

**Keywords:** public health emergency, public policy, policy evaluation, qualitative, quantitative

## Abstract

Policy is an important support for risk society to prevent and resolve crises. Based on the content analysis of the policy text and PMC-Index model, this paper takes texts of 327 public health emergency response policies (PHERP) at the central level in China from 1989 to 2022 as the analysis object, designs an indicator system, and combines qualitative and quantitative methods to evaluate the existing policies. The results of content analysis indicate that current policy focuses on emergency rather than preventive control, the main policy-making and issuing authority is the Ministry of Health and policies are mostly issued in the form of notice. The PMC-Index of ten selected policies is all ranked above acceptable, which means that the overall quality of policy text is relatively high. However, the PMC-Surface shows that there is still considerable variability in the scores of the main indicators for each policy. The top three main scoring indicators are policy nature, policy evaluation and policy instrument, while the bottom three are policy time, policy release agency and policy target groups, which reminds us that the design of policy text can still be improved in terms of optimizing policy time, policy issuing institutions and expanding policy target groups. In response to these problems, this paper puts forward six suggestions for optimization.

## 1. Introduction

In January 2020, the World Health Organization (WHO) listed the outbreak of COVID-19 as a “public health emergency of international concern” (PHEIC) which refers to unusual events determined according to the “Provisions of the International Health Regulations”, highlighting the fact that the international spread of an event can result in a disease that poses a public health risk to the world and, therefore, requires a coordinated international response [1]. As of 24 August 2022, the cumulative number of confirmed cases of COVID-19 reported worldwide exceeds 500 million and the cumulative number of deaths exceeds 6 million. In July 2022, WHO once again identified monkeypox outbreaks in many countries and regions as public health emergencies of international concern. Since 2003, WHO has declared seven international public health emergencies. Public health crises are latent in the realm of everyday life, erupting in uncertain time and space situations, and the hazards can spread under certain conditions, creating a chain reaction that can cause serious social harm [2,3].

In China, public health emergencies are defined as major epidemics of infectious diseases, mass illness of unknown origin, major food and occupational poisoning and other events seriously affecting public health that occur suddenly and cause or may cause serious damage to public health [4]. From the above two definitions, we can see that public health emergencies are characterized by wide spread, diverse causes, strong harm and short reserved crisis response time [5]. As a result, the prevention, control and emergency response capabilities for such events are more demanding and the scope of crisis management is broader.

With the interplay of spatial and temporal scenarios in the process of globalization, the interaction of individuals with other individuals and the world has become more frequent, and public health crises that have not been completely prevented continue to emerge, making the risks to public health seemingly ubiquitous [6]. This just falls into the logic of the “social properties of risk” proposed by Ulrich Beck [7]. Risk society is a kind of insecurity and system control contingency brought on by globalization itself. Beck’s joking language, “poverty is hierarchical, and smog is democratic” [7], reminds us that risks have unprecedented intensity and breadth [8]. COVID-19, which has spread to more than 200 countries and territories worldwide, has proved to us that the impact of such events on human health, life and social development is profound and far-reaching, and once the risks are made public, they have the rigidity and intensity of politics [7]. Applied to the prevention, control and management of public health emergencies, this summary can be interpreted as that although public health emergencies challenge the productive order of human life, this widespread rigidity and intensity can also go some way towards breaking down social antagonism, suppressing established group conflict and providing justification for the search for a greater sense of identity and social solidarity. In this process, it is not only a test of national governance and crisis response ability, but also a great opportunity to increase citizens’ recognition and trust in the government. The possibility of reversing the negative function of risk to promote social identity and social cooperation is constructed on the source of risk—decision making [9] and the ongoing process of modernization is driven by the reorganization and change of mechanisms and institutions. It is thus clear that decision-making is of great importance in anticipating crises, resolving them and reducing risks. Giddens, another scholar concerned with risk society, shares Beck’s understanding of the characteristics of risk, which he also sees as social in nature—as a human social relationship—and that risk arises from the continuing disembedding of modern society, two features of which are “symbolic mechanisms” and “expert systems” [10]. When society migrates along the path of globalization, and its complexity gradually increases and people can no longer grasp it on the basis of kinship relations such as blood and geography, the knowledge based on geographical characteristics that they originally relied on is replaced by expert systems with standardized and specialized characteristics, when actors rely not on this standardized knowledge but on trust in science and the community of scientists as a whole. The essence of the expert system is an escalation of the social divide, with which the individual is exposed to a high degree of social risk, but the individual can live with risk, not because the individual lacks the rational perception of risk and the ability to avoid it, but because the individual chooses to overcome it and live together with others based on social choices. Trust arises when the individual, having carried out social action through social instincts, verifies inductively that his previous social instinctive trust is not only an emotional confidence, but also that his choice has turned out to be rational, at which point the reintegration is completed and trust arises [8].

The enlightenment of this theory in dealing with public health emergencies such as COVID-19 is that policies and society are not helpless when facing risks. As mentioned above, risks still contain opportunities; firstly, we need to recognize that responses to unsafe, risky events in society require a shared understanding of them, and that a major source of this understanding is decision-making in society. Decisions are often communicated to the public in the form of institutions and policies, and as a more operational and socially relevant approach, policy creates a common basis of perception between policy-makers and actors, and this basis is constantly interpreted and changed in the interaction between them. A standard is thus established that reinforces the public’s perception. However, there is a gap between standards and rationalization, and science also has limitations and is not effective in identifying and correcting the minor aberrations in the distribution of social power and internal misconduct. Therefore, we should always review the existing standards to correct and amend the parts that are inconsistent with the actual development.

As a form of decision-making expression, policy plays an important role in preventing and reducing risks. At the same time, people who trust the government’s decision-making driven by the group’s social instincts will once again embed their trust in the government after verifying the rationality of this social trust in practice [11]. Almost every country and region will issue corresponding policies for the prevention of and response to public health emergencies, but because the formulation of standards is limited by the development of society and the development of scientific knowledge on public health prevention and control, it is inevitable that the content of policies will lag behind, resulting in deviations between the content and actual operations. Regular evaluation and revision of existing policies is an effective way to ensure that policies can reasonably respond to public health crises [12,13,14] and is also the embedded core of government trust building. However, most of the current evaluations of PHERP are based on the implementation process and effectiveness of the policy, examining the effectiveness of the policy after it has been implemented. For example, comparison of different cities’ response speed to policies [15], the impact of financial policies issued in public health emergencies on the financing of private enterprises [16] and exploring the legality of accessing the privacy of citizens’ location information for COVID-19 outbreak prevention and control policies [17]. In terms of assessment methods, theoretical analysis [18], empirical research [16,19,20] and causal inference [21] are mostly used. There are few scientific quantitative assessments of the comprehensiveness, consistency and rationality of the policy text itself. The content of PHERP texts is an important basis for policy implementation: What methods can be used to evaluate policy texts? How should the assessment system be structured? What is the quality of the content of current policy texts? Is the design of policy texts scientific? Is the content of policy texts forward-looking? Does it cover the full range of needs in a public health emergency? Is the policy content consistent? These are among the most important questions we need to answer in the prevention and emergency response of public health emergencies [22].

Against this background, this paper will take the texts of China’s PHERP over the years as the research object, introduce two relatively objective analysis tools, attempt to establish a quantitative evaluation index system for PHERP and combine qualitative analysis methods to focus on the strengths and weaknesses of existing policy texts, so as to put forward some suggestions for improving the design of subsequent policy texts.

Next, the content of this paper is roughly divided into four parts: The second part is the carding and evaluation of the existing research results of PHERP evaluation; the third part is a detailed research design of this paper; the fourth part mainly discusses the results; the fifth part is the conclusion and summary of this article.

## 2. Literature Review

### 2.1. Theory and Practice of PHERP

From the perspective of release time and policy purpose, PHERP can be roughly divided into two categories: emergency preparedness and disposal [23,24]. The focus of existing research findings on policy tends to be on the formulation and implementation of emergency disposal policies.

Current research on PHERP has focused on the classification of policies and the exploration of policy effects. In general, scholars have paid attention to macro-social policies and individual protection policies at the micro level in response to public health emergencies. Macro-level policy research involves fiscal and tax policies [25,26], employment policies [27], resumption of work and production policies [28], criminal prevention and control policies [29,30], etc.

At the micro individual level of public health events, more scholars pay attention to the policy content of personal information and privacy protection; in order to detect and combat outbreaks in a timely and accurate manner, the government needs to have access to citizens’ personal information such as their travel and interpersonal history, but there is also a risk of citizens giving up their personal privacy information, and there is still a gap between existing personal information protection and policy practice in China [31]. The main problem lies in the unclear policies and regulations on the boundary of individual rights and obligations in the process of handling public health emergencies, and the lack of legal and policy system support for the protection of citizens’ privacy [17], and deviations in policy formulation not only limit the government’s ability to control outbreaks of infectious diseases but also lead to a lack of public confidence in the government [32]. After comparing the policies and regulations on the use of personal data in Europe and the United States, Li Yifei and Wang Xiezhou et al. found that the policies and regulations on the collection and processing of personal data in the European Union and the United States are worthy of our reference [33].

### 2.2. Research Practice in Policy Evaluation

Policy evaluation is a systematic science that assesses the strengths and weaknesses of policies from the perspective of formulation, change and adjustment, and implementation [34]. Key evaluation approaches include qualitative analysis and quantitative assessment. After sorting out China’s emergency response policies for public health emergencies in the past decade, Sun Mei found that China’s emergency response policies for public health emergencies were gradually refined from 2003 to 2014, but some policies still lacked operability [35]. Hu Guoqing et al. used a stratified sampling method to randomly select 66 Chinese municipalities, and the results of a questionnaire survey on their emergency plans for public health emergencies showed that the existing public health emergency plans were of low quality and more than 50% of the municipalities lacked emergency planning regulations [36]. After collating existing legislation and policies on public health emergencies, Chen Wei concluded that the existing legislation has certain omissions and deficiencies in the stages of incident reporting, notification and announcement, control, treatment and supervision and management [37].

Although qualitative evaluation is useful for policy analysis and summaries, some scholars argue that this type of evaluation is highly subjective in terms of evaluation indicators and that the evaluation results are not objectively referenced [38]. In recent years, academics have attempted to introduce quantitative thinking into the field of policy evaluation. Wu Kechang and Wu Chuhong used the Cox risk proportional regression model of the event history analysis method to examine the relationship between leaders’ education background, urban industrial enterprise ownership and patient healing capacity and the speed of policy response to public health emergencies, and it was found that there was a positive relationship between them [15]. After reviewing the public’s emotional feedback on the delayed start of school policy in social media data, Wen Hong and Zheng Hong believe that under the severe epidemic situation, most of the public are generally negative about the delayed school opening policy, but there are differences due to the impact of the epidemic, the level of economic and education development and other factors [20].

In the field of quantitative research on policy texts, Cai Luming and Hu Xiangming found that the existing public health emergency management policies are “unbalanced” in many dimensions after analysing the policy samples with the qualitative data analysis software, Nvivo [39]. Pei Junliang et al. built a framework model of a government public health emergency information reporting system with preconditions, three stages and two supports by using the cluster qualitative content analysis method [40], or analysis PHERP through the qualitative content analysis approach under the two-dimensional analysis framework of “policy objectives–policy instruments” [41,42].

### 2.3. Summary of Policy Research Review

Existing studies have extensively discussed policy types, policy characteristics and the policy effects of public health emergencies and have achieved fruitful results, but the following shortcomings may exist: First of all, at the level of research objects, existing studies have conducted a relatively full discussion on various types of PHERP, but PHERP should also include ex-ante prevention and ex-post recovery and reconstruction, and existing studies lack attention to public health emergencies’ pre- and post-response policies. At the same time, research on policy quality focuses on outcome effect tests and neglects evaluation studies on the quality of policy texts.

Looking at the analysis results of other types of policies, we find that scholars have made new progress in the use of quantitative analysis tools for policy texts. On the one hand, scholars make full use of natural language processing tools, which can transform text data into numerical data, analyse and process text semantic information [43] and extract high frequency vocabulary from the text and condensing key policy messages [44]. This method, currently widely used in the field of policy text analysis [45,46,47,48], usually requires the use of intelligent text analysis software such as Python, Rost Content Mining 6.0 [49,50,51,52]. On the other hand, scholars have introduced the Policy Modeling Consistency Index model into the field of quantitative policy evaluation, and its strength lies in the fact that the model itself is designed for the content of policy texts, at the same time, the model can not only quantitatively evaluate a single policy, but also conduct comparative analysis on multiple policies [53]. As a result, experts in several policy areas have used the model to fully quantify policy texts [54,55,56].

Given the lack of application of these methods in the field of PHERP evaluation and the high adaptability of PHERP texts to natural language processing tools and the PMC-Index model, in the following parts, two natural language processing tools, Python, Rost Content Mining 6.0 and the PMC-Index model are introduced into the analysis process to reasonably establish a comprehensive evaluation system for PHERP and conduct a multidimensional analysis on the scientificity, rationality, consistency and comprehensiveness of the contents of PHERP in China.

## 3. Research Design

### 3.1. Content Analysis of Policy Text

Policy texts are by their very nature collections of words, numbers, punctuation and other symbols that combine to reflect the will and intentions of public authorities such as the government, as well as being a reflection of government action [57]. Objective textual records and expressions provide us with the possibility of qualitative and quantitative analysis of textual content. In this paper, we will use natural language processing tools to analyse the collected policy texts in terms of sub-word statistics and semantic relationship networks, so as to help us gain a comprehensive understanding of the existing policy features, trends and inherent relationships of PHERP.

**Figure 1:**
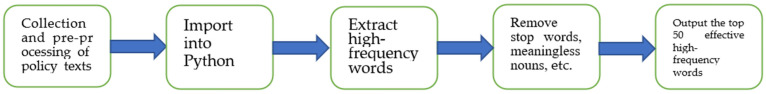
Process of high-frequency words’ statistics.

The content analysis of the policy text in this paper is divided into two main parts. The first part is a high frequency vocabulary count using Python software. There are five steps in the specific operation process (see Figure 1): The first step is to convert the collected texts into a format that can be recognised by the processing software; the second step is to import the collated text to be analysed into the Python software; the third step is to use the adaptation code and Chinese word segmentation package to perform word segmentation and word frequency statistics on the text; the fourth step is to filter and process the analysis results and remove the stop words, the frequently used nouns, quantifiers and verbs which cannot reflect the text features and have little to do with the analysis; the fifth step is to sort out the top 50 effective high-frequency words. Through these high-frequency words, we can preliminarily describe the characteristics and development trends of China’s PHERP and provide a basis for the selection of indicators for subsequent model building.

**Figure 2:**
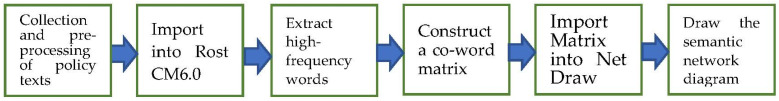
Process of semantic network analysis.

Semantic network analysis is the second part of content analysis. The software tool used is Rost Content Mining 6.0. Similar to the previous analysis method, semantic network analysis also requires word segmentation and word frequency statistics for text content, but what makes this method different from the previous ones is that, after filtering out the high-frequency words, it also allows for the construction of a co-occurrence matrix to derive the focus and centre of attention of the policy text as well as the associations between key words and the interpretive context surrounding them [58]. The specific steps are as follows (see Figure 2): First, extract the title of each policy text, and sort out the text content according to the provisions of the software; next, import the text to be analysed into the software and select the semantic network analysis function; finally, the co-word matrix is constructed after extracting high-frequency words, and then the matrix is imported into Net Draw software to form a visual semantic relationship network diagram, which allows us to understand the associations between high frequency words and the strength of the links between them.

### 3.2. PMC-Index Model

PMC-Index calculation refers to the analysis of the level of internal consistency of the policy by measuring the PMC-Index through several indicators. With the model we can quantitate the policy texts and evaluate them. Using a three-dimensional visual spatial construction method, a PMC-Surface is created to present the overall status of the policy and the specifics of each individual policy to evaluate the strengths and weaknesses.

**Figure 3:**
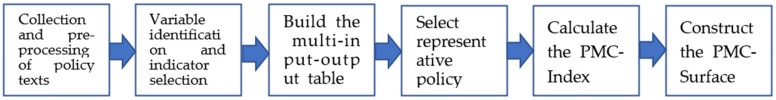
Process of application of PMC-Index and PMC-Surface.

In recent years, the PMC-Index model and PMC-Surface have been widely used in the analysis of various policy texts [59], offering the possibility of quantitative evaluation of policy texts. The model can be used to assess the internal consistency of multiple policies and to objectively and visually evaluate the strengths and weaknesses of policies along a number of indicator dimensions in a four-step process (see Figure 3):

(1) Selecting indicators and identifying parameters

Before evaluating the policy, we must collect as many valid relevant variables as possible from all policies and classify the collected variables, eventually selecting a number of primary and secondary variables. In the PMC-Index model, all parameters are set in binary 0 and 1, which can ensure that the weight of each secondary variable is the same.

(2) Building the multi-input–output table

Multi-input–output table is a data analysis framework which results from the multi-dimensional measurement of variables and is composed of primary and secondary variables. In the current study, all secondary variables had equal weights. When the policy text contained or incorporated a secondary variable, the parameter value of that secondary variable was marked as 1; otherwise, it was marked as 0.

(3) Calculating of the PMC-Index

In calculating the PMC-Index of policy texts, we first construct main and sub-variables based on research findings in the previous PHERP literature, combined with policy text characteristics, with the Formula (1):

X∼N [0, 1](1)

Build a multi-input–output table and assign specific stated values to the secondary variables based on the results of the text analysis and the binary method, with the Formula (2):

X = {XR: [0~1]}(2)

Combining the assignment of secondary variables to calculate the value of primary variables, the formula became (3) and (4):

(3) $$X_{i}=\sum_{j=1}^{n}\frac{X_{i,j}}{n}\;\;\;i=1,2,\ldots ,10$$

(4) $$\mathrm{PMC}-\mathrm{Index}=\sum_{i=1}^{10}X_{i}$$

*i* = main indicator; *j* = sub-indicator; *n* is the total number of the sub-indicator.

### 3.3. Data Sources for Policy Texts

As the highest political will of the state and the authoritative carrier for emergency prevention, control and emergency management, the central-level public health emergency response policy can clearly reflect the realities, historical changes and complexities of public health emergency response. Therefore, this paper selects central-level public health emergency response policies issued by the CPC Central Committee, Central Government, various ministries and national administrative organs as the research object and uses core search terms such as “public health emergencies”, “emergencies”, “public health”, “epidemics”, “emergency policy” and “public health policy” on the Chinese government website, National Health and Wellness Commission, Chinese Center for Disease Control and Prevention and Peking University Laws and Regulations Database. After intensive reading of the title and content of the policy text, the policy texts with little relevance to the topic and texts, which are only technical guidance on specific matters such as equipment installation in the search results, were eliminated, and 327 valid policy texts were finally selected for analysis. The content of the screened policy text is highly relevant to the prevention and response of public health emergencies, and the text data has high internal validity, which can better serve the interpretation and research of this theme. In addition, the contents of the texts are documented in objective texts and are publicly released by authoritative bodies, thus providing a good level of reliability in the research sample.

### 3.4. Data Description

#### 3.4.1. Types of Public Health Emergencies Response Policies

Based on the criteria of policy release time point, policy purpose and policy function, policies related to public health emergencies can be divided into two main categories: emergency preparedness beforehand and emergency response during and after the event. Emergency preparedness mainly includes emergency team construction, system construction, material storage, standard establishment, regulation improvement, emergency skills training, public health talent training, etc. The emergency response during and after the outbreaks of events focuses on the areas of information reporting, on-site response division, information dissemination, treatment plans, market order, resumption of work and production, and the job market. Through sorting out and statistics, the current policies are mainly issued in 17 forms, including laws, measures, emergency plans, notices, outlines, opinions, decisions, plans, regulations, emergency notices, regulations, procedures, key points of work, announcements, guidelines, norms and plans. Among them, the most commonly used form of policy release is notice, accounting for 76.5% of the total. There are five legal texts with the highest effect, namely the law of the People’s Republic of China on the prevention and control of infectious diseases (1989), the law of the People’s Republic of China on the prevention and control of infectious diseases (2004), the law of the People’s Republic of China on emergency response (2007), the law of the People’s Republic of China on the prevention and control of infectious diseases (2013) and the vaccine management law of the People’s Republic of China (2019).

#### 3.4.2. Number of Public Health Emergency Response Policies

The earliest policy issued by China in response to public health emergencies was the Law on the Prevention and Control of Infectious Diseases, adopted by the Standing Committee of the National People’s Congress in 1989, whose main objective is to prevent, control and eliminate the occurrence and prevalence of infectious diseases and safeguard human health. The introduction of this law filled a legislative gap in the prevention and control of infectious diseases at the time and established the principle of classifying and managing infectious diseases and a system for the notification and publication of epidemics. Over the next 33 years, China has issued 326 related policies. In terms of the number of policies issued in a single year, 2020 ranked first with 63 policies, with most of the policies issued in this year focusing on the theme of the control of the COVID-19.

The concept and extension of “public health emergencies” was first formally introduced in the “Regulations on Public Health Emergencies”, issued by the State Council in 2003. In addition to the types of infectious diseases specified in the law on the prevention and control of infectious diseases, the regulations also include mass diseases of unknown causes, major food and occupational poisoning and other events that seriously affect public health, such as public health emergencies. Moreover, the procedures and norms for prevention and emergency preparedness, reporting and information release, and emergency handling of public health emergencies have been clarified in the regulation.

**Figure 4:**
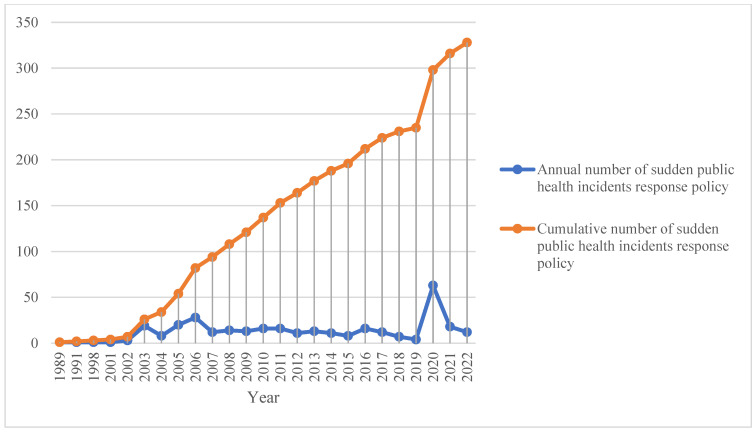
Annual and cumulative number of entrepreneurship policy.

As we can see from Figure 4, there are two large jumps in policy volume in 2003 and 2020, and in the context of their respective policy development, the uptick in policy number at these two points is largely attributable to the two public health emergencies of SARS and COVID-19. In response to the sudden onset of a highly contagious and widespread epidemic, a large number of policies have been issued, including many in the form of “emergency notifications”. However, after the concentrated outbreak of the epidemic, the number of policies issued in a single year decreased significantly.

#### 3.4.3. Agencies and Department Issuing PHERP

The data that have been collated and counted indicate that between 1989 and 2022, 44 departments and agencies have issued guidance and response policies for public health emergencies. The largest number of articles was issued by the Ministry of Health (merged with the Population and Family Planning Commission in 2013 to form the National Health and Family Planning Commission), with a total of 127 articles issued between 1998 and 2013.

In general, the main body of public health emergency response policies is the health administrative departments including the Ministry of Health, the National Health and Family Planning Commission, the National Health and Health Commission (which was integrated by the responsibilities of the National Health and Family Planning Commission in 2018), and a total of 262 documents were issued. The number of joint documents issued by different departments and institutions is 60, the “Notice on printing and distributing the work guide on coordinating the prevention and control of COVID-19 epidemic and ensuring the supply and price of ‘vegetable basket’ products” is the policy with the largest number of joint issuers among all policies, with a total of 11 departments including the Ministry of Rural Agriculture, the National Development and Reform Commission, the Ministry of Finance, the Ministry of Natural Resources, the Ministry of Ecological Environment, the Ministry of Transport, the Ministry of Commerce, the National Health Commission, the State Administration of Market Supervision, the China Banking and Insurance Regulatory Commission, the CSRC and 11 other departments participated in policy formulation and release.

### 3.5. Variable Selection and Parameter Identification

According to the Omnia Mobilis hypothesis [60], selecting as many evaluation indicators as possible is the first step to do a good job in evaluation research of PHERP. Combining the results of existing research and textual analysis, the following ten main indicators have been selected: X_1_ Policy nature, X_2_ Policy instruments, X_3_ Policy time, X_4_ Policy field, X_5_ Policy function, X_6_ Policy release agency, X_7_ Policy action mode, X_8_ Policy target groups, X_9_ Policy evaluation and X_10_ Policy disclosure. The results of the selection of primary indicators, secondary indicators and the rationale for their selection are shown in Table 1.

### 3.6. Selected Assessment Objects

Considering the large total number of policy texts and the limited space of the article, this paper takes into account the type and form of policies and the content of policies on the basis of the results of the content analysis of policy texts and selects representative policies of different periods for quantitative assessment (see Table 2).

### 3.7. Construction of PMC-Surface

After obtaining the values of the indicators, constructing a surface of the PMC-Index can make the objective figures vivid and three-dimensional. The realization of this result relies on the construction of the PMC matrix. Based on the values of the nine main indicators (we eliminate X_10_ given that the matrix is balanced and symmetrical and X_10_ has little impact on the variability of the results), we create a 3 × 3 square matrix and use Formula (5) to prepare a PMC-Surface of ten policies, and the process is shown in Figure 3:

(5) $$\mathrm{PMC}-\mathrm{Surface}\;=\;\left[\begin{array}{ccc} X1 & X2 & X3\\ X4 & X5 & X6\\ X7 & X8 & X9 \end{array}\right]$$

## 4. Results and Discussion

### 4.1. High Frequency Vocabulary Statistics

Statistical results help us to understand the main content of the policy texts objectively. After segmenting the content of the policy text, remove the stop words that cannot reflect the text characteristics and the nouns “China” and “Year” that are not significant to the analysis results, and finally sort out the top 50 effective high-frequency words. See Table 3:

High frequency words such as “health”, “emergency” and “management” suggest that the focus of the existing public health emergency response policy is the emergency disposal of public health emergencies, which can ensure public health. The words “disease”, “infectious disease”, “prevention” and “prevention and control” mean that the prevention and control of public health emergencies in China at this stage mainly start with infectious diseases. In contrast, the frequency of the terms “public health” and “event” shows that “public health emergencies” appear less frequently than infectious diseases, indicating that China’s early understanding of public health emergencies was limited to infectious diseases, and, in terms of policy facts, the concept of “public health emergencies” was not clearly defined in China until 2003. The terms “epidemic”, “information”, “monitoring” and “reporting” indicate that China’s existing response policies have devoted more attention to the development of monitoring and reporting systems for epidemic information. The vocabulary in lines ten to fourteen indicates that the organisation of the management of prevention, control and emergency response to public health events is set out in the policy text as the health administration, while health care institutions are primarily responsible for the treatment of patients’ conditions. “Indicators”, “standards” and “norms” showcase China’s standardized scientific prevention and emergency exploration.

### 4.2. Semantic Network Analysis of PHERP

**Figure 5:**
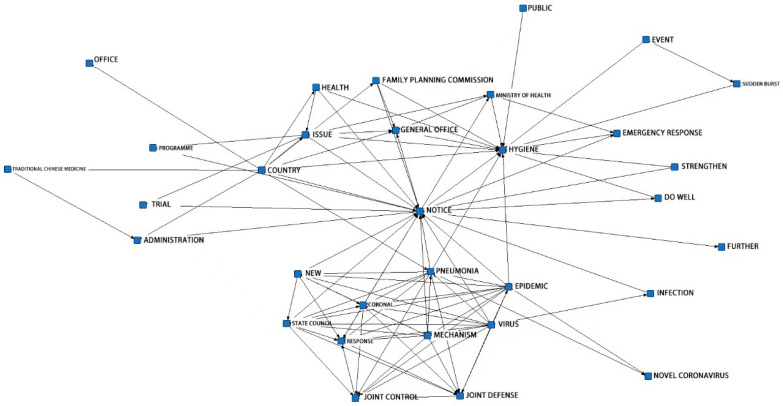
Semantic network analysis result.

As shown in Figure 5, nodes such as notification, health, pneumonia and so on are more central, and the connections with other nodes are denser, of which notification is at the core of the semantic network. This result is consistent with the previous statistics on the form of policy issuance. The Ministry of Health is an important department that formulates and publishes PHERP. In addition, the lines between the nodes in the lower area of the chart are more intertwined than in the other parts, indicating a higher correlation between these nodes and a higher frequency of occurrence in all policy texts. In combination with the basic features of the policy, these nodes come from China’s policies on dealing with novel coronavirus pneumonia infection in 2020. During this period, China established the State Council’s Joint Prevention and Control Mechanism for COVID-19, which was formed by a number of ministries and commissions at the central government level and issued policies on matters previously under the jurisdiction of various central government departments, such as epidemic prevention and control, medical treatment, scientific research and research, publicity and foreign affairs; therefore, the policies are highly integrated and cross-cutting, and then appear in the network diagram as a densely woven network and highly interrelated nodes.

### 4.3. Policy Evaluation of PHERP Based on PMC-Index Model

#### 4.3.1. Building the Multi-Input–Output Table

After 10 main variables and 39 sub-variables have been selected, we specify parameter identification using binary as a criterion. If the selected assessment sample meets the requirements and conditions described by a sub-indicator, the corresponding sub-variable parameter will be scored as 1, otherwise it will be scored as 0 (see Table 4).

#### 4.3.2. Calculating the PMC-Index

After assigning values to each policy against the criteria and content of each secondary variable, this paper first calculates the scores of the primary variables based on the sub-indicator scores of each policy and finally derives the PMC-Index of the policy according to the calculation formula (see Table 5). The ten main indicators have a full score value of 10, so the calculation can be divided into four levels: 8–10 can be considered Optimal, 6–7.99 is Good, 4–5.99 is Acceptable and a score below f4 is identified as Poor (see Table 6).

#### 4.3.3. Detailed Description of PMC-Index

In general, the quantitative results of the PMC-Index for the ten representative policies are all above 4, with two policies rated Optimal, six policies rated Good and the remaining two all rated Acceptable (see Table 6). This indicates that the current PHERP follow the principles of science, reasonableness and operability, that the policies are adjusted in a timely manner according to the needs of public health protection at different times and that the synergy between policies is at a high level.

**Figure 6:**
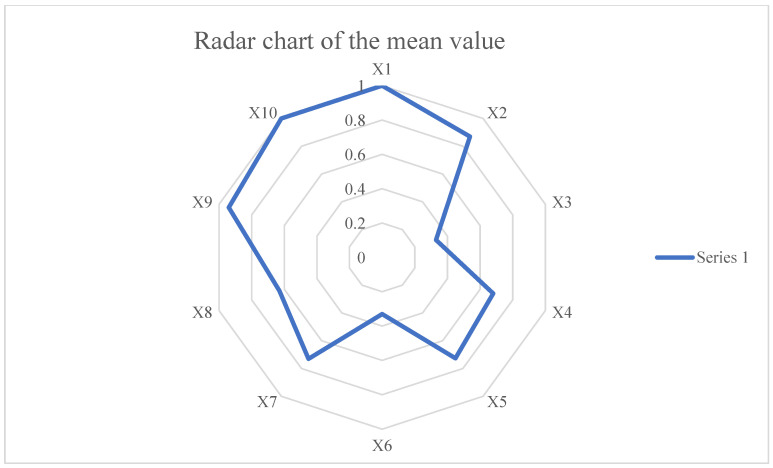
Radar chart of the mean value.

In order to visualize the scores of each main indicator, we have created a mean radar chart (see Figure 6) which is irregularly shaped, with the highest score for policy nature (X_1_), the lowest score for policy time (X_3_) and policy release agency (X_6_) among the ten main indicators. It can be seen that the scores of various indicators of the policy are still unbalanced, which reflects that there are still some weaknesses in the PHERP and provides an objective basis for the formulation and revision of policies in the future. The specific discussion is as follows:

As shown in Figure 6, policy nature scores perfectly, which indicates that policies are able to balance the nature of prediction, regulation, advice and guidance.

The average scores of X_2_, X_5_, X_7_ and X_9_ are all above 0.7. This result means that the design and resource allocation of PHERP is relatively balanced in terms of policy instruments, policy fields, policy functions, policy action modes and policy evaluation. Policies comprehensively use supply-based, demand-based and environment-based policy tools in a broader manner. The policy takes into account eight functions, such as medical treatment guidance, popular science publicity and education, prevention points before the event, recovery and reconstruction after the event, and is able to flexibly use three modes of action, such as coercion, service and motivation. In the design of text content, the goal is clear, the basis is sufficient, the scheme is reasonably designed, the planning is detailed and the rules of responsibility and power exercise are clearly explained. Therefore, the score of policy function and evaluation is high.

The policy time X_3_ and policy release agency X_6_ ranks tenth, with 0.33. The shortcoming of this item come from the lack of clarity in the content of the policy text regarding the effective time frame of the policy. Most of the texts lack a time-bound planning of the policy objectives and generally show a single period of planning and assessment, which is not conducive to the efficient achievement of the policy objectives.

The quantitative result of the policy releasing agency matches the number of joint issuances in the data description. The linkage between the various issuing bodies at all levels is not yet close, with a single body accounting for 82% of issuances, and this result has the potential to lead to a separation of institutional responsibilities in the policy implementation process, thus affecting the effectiveness of policy implementation.

The top three quantitatively rated policies are P_10_, P_3_ and P_4_ (see Table 6). The first two policies were both issued by the State Council, P_3_ and P_4_ are both contingency plans and P_10_ is the National Health Plan, just released in 2022.

As the policy with the highest score among the ten policies, P_10_ obtained full marks in all other indicators except X_3_ and X_6_, which are generally not high in average scores. From its title, we can see that this is a comprehensive policy at the macro level, integrating the prevention, control and response of health emergencies into the national health plan. The policy covers economy, politics, education, social services, social environment and other aspects. In addition to the supply-based policy instruments such as fund guarantee and public health talent team building, the policy also proposes to strengthen international cooperation in the field of public health and the construction of a public health rule of law system. In addition, compared with the other nine policies, the reason for the highest score of P_10_ is that the policy text clearly proposes to innovate the social mobilization mechanism and include enterprises, non-profit organizations and all citizens in the prevention, control and disposal of public health emergencies. On the one hand, enterprises, social groups, non-governmental organizations and other organizations can help prevent and control diseases. On the other hand, by joining in the prevention and control activities organized by government departments, the above-mentioned subjects can participate in the process of social governance, which can not only practice the concept of multi-body common governance but also deepen national identity and promote social unity [65].

The shortcoming of the policy lies in the failure to specify in the text the phased assessment time frame for the realisation of the plan. The achievement of the policy effect not only relies on the scientific design of the policy text but also requires the effective cooperation of the policy implementation. Therefore, the stipulation of policy time should not be limited to the length of the policy effectiveness but should also pay attention to the design of the phased implementation assessment scheme for policies.

P_3_ and P_4_ are emergency plans for public health emergencies. The former is the “National emergency plan for public health emergencies” issued by the State Council in 2006, and the latter is the “National health emergency plan for natural disasters” issued by the Ministry of Health in 2009.

From the perspective of policy jurisdiction, P_3_ is the emergency reserve for all types of public health emergencies nationwide, and P_4_ is the emergency plan for health emergencies after natural disasters. In terms of effectiveness level, P_3_ is the superior law of P_4_.

From the quantitative evaluation results, the scores of X_1_, X_2_, X_3_, X_5_, X_6_, X_7_ and X_9_ of the two policies are the same, and the differences are mainly reflected in the scores of X_4_, X_5_ and X_8._ In terms of the X_4_ policy field, the score of P_3_ is lower than the average score of the main indicators. The policy contents of P_3_ and P_4_ involve economic, political and social environments. However, there is little mention of the emergency plan in the field of education and no detailed plan for the handling of public health events within educational institutions and the emergency response of educational institutions after the outbreak of public health emergencies; at the same time, the administrative department of education is not included in the coordination mechanism. P_4_’s performance in X_5_ policy function is inferior to P3’s, and the main gap comes from the vacancy of the policy’s function of stabilizing social order. Public health emergencies will have a certain impact on market supply, price level, social security, etc., so it is very necessary to embed order maintenance clauses in the policy content. The practice of fighting against COVID-19 shows that the basic living guarantee of the masses during the epidemic is equally important. Therefore, in 2022, the Ministry of Agriculture and Rural Affairs, the National Development and Reform Commission, the Ministry of Finance and other ministries and commissions jointly issued the work guide for coordinating the prevention and control of COVID-19 epidemic and ensuring the supply and price stability of “vegetable basket” products to maintain citizens’ living order. P_3_ and P_4_ policy texts are rich in guiding the work and life of government departments and people, however, the common shortcoming is that there is no mention of the code of conduct and policy safeguards that social groups, such as non-profit organisations, should have in response to emergencies. In fact, volunteer organizations and rescue organizations have played an important role in every emergency. Policy is not only the practical guide to prevent and respond to emergencies, but also the embodiment of social values. Therefore, the formulation of public policy should not only be problem-oriented but also take into account value orientation [66].

The three policies at the bottom of the list are P_9_, P_7_ and P_8_, which are professional classification policies issued by various ministries and commissions, focusing on emergency response team building, public health prevention and treatment capacity building, and food safety risk detection. They scored the same on the four primary indicators X_1_, X_2_, X_3_ and X_6_. Among them, the score of X_2_ is lower than the average. The use of demand-based policy instruments is zero in P_9_ and P_7_, while the use of supply-based instruments is missing in P_8_; overall, there is some bias in the proportion of policy instrument use. Otherwise, all three policies performed poorly in terms of policy field, policy function, policy mode of action and policy target group. On the one hand, these shortcomings are limited by the professionalism and non-comprehensive nature of the policy content, but at the same time, there is still room for improvement in the design of the policy content. Taking the scores of X_4_, X_5_ and X_7_ of P_8_ as an example, although the policy objective is to risk testing for food safety, the content of the policy still needs to consider the work arrangements in economy, education, social services, social environment and other aspects. The restricted nature of the policy content leads to a limited policy function. The policy function of P_8_ is only manifested in three aspects: prevention in advance, event monitoring and reporting and emergency system construction. However, in fact, interspersing the policy content with science education and regular food safety risk prevention and control measures will not conflict with the achievement of the policy objectives; otherwise, issuing policies to achieve a single objective will inevitably lead to policy redundancy. Furthermore, the traditional Chinese policy implementation method relies on the vertical mechanism of administrative power, which can ensure the effective implementation of policies and prevent the occurrence of “executive vacancy”, however, this mode may also lead to the policy action mode being trapped in the “mandatory type”, which obviously runs counter to the concept of “multi-agent collaborative governance”, and is not conducive to the mobilization and use of social resources during the sudden crisis.

#### 4.3.4. PMC-Surface of Four Policies

The PMC-Index results for the six policies, P_3_, P_4_, P_7_, P_8_, P_9_ and P_10_, have been discussed in detail in the previous section, and the following paper will analyse P_1_, P_2_, P_5_ and P_6_ in three dimensions by means of constructing a PMC-Surface.

**Figure 7:**
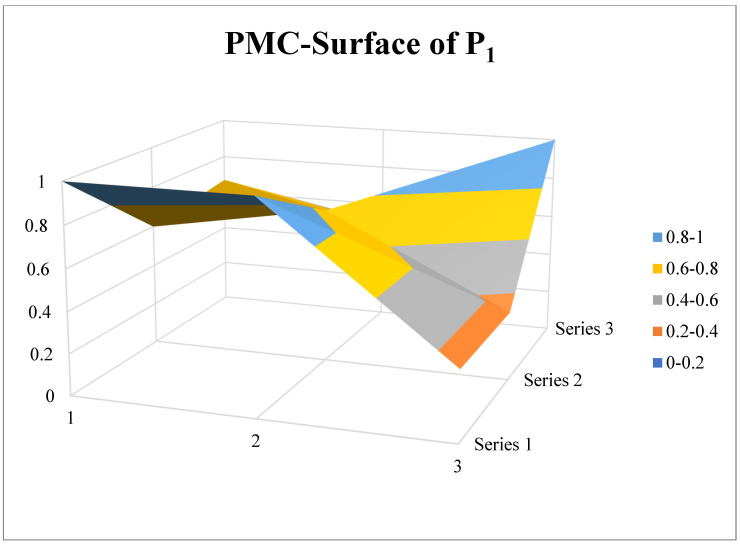
PMC-Surface of P_1_.

P_1_ is the implementation measures of the law on the prevention and control of infectious diseases issued by the State Council of China in 1989, which ranks sixth in PMC-Index. From the title, it can be seen that it is a supporting implementation measure of the law on the prevention and control of infectious diseases, and its policy objective is to implement the law with the highest effectiveness. From the surface chart (Figure 7), we can see that there is a large imbalance in the indicators at all levels of the policy. The concave part in the figure is mainly concentrated in the upper and lower corners, which reflects the low scores of X_3_, X_6_ and X_8_ in the PMC-Index results. In addition to the common low scores of X_3_ and X_6_ of all policies, the policy target groups of P_1_ are limited to government departments and the public. At the same time, the scores of X_4_ and X_7_ are lower than the average value because the policy does not involve the economic and social services field, and the implementation of the policy is limited to coercion and incentives.

**Figure 8:**
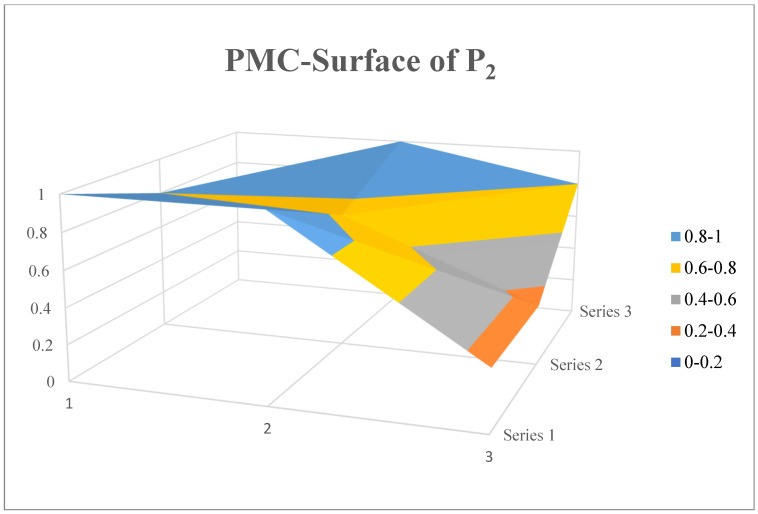
PMC-Surface of P_2_.

P_2_ is the notice on printing and distributing the outline of the Tenth Five Year Plan for national prevention and control work issued by the general office of the Ministry of Health in 2001. Its policy objective is to actively prevent and control major and key infectious diseases endangering public health through reform, establishment and improvement of the disease prevention and control system and improve the emergency response and handling capacity of public health emergencies. Compared with the above figure, the curved surface of P_2_ (Figure 8) has less undulation, and the concave part is mainly concentrated in the lower right corner, that is, the policy time and the policy issuing institution. P_2_ is an outline for the construction of health prevention and control over the next five years. In the text, policy makers detailed the specific objectives of future work from six aspects: system reform, infectious disease prevention and control, vaccine preventable infectious disease control, endemic disease and parasite prevention and control, chronic non-infectious disease prevention and control and rural health construction, however, in contrast to the clear work objectives, the policy text does not include a phased assessment plan for the achievement of the work objectives, so that there is a lack of effective supervision and guidance for the achievement of the policy objectives. In addition, there are many aspects of the policy that exceed the authority of the Ministry of Health, such as intergovernmental cooperation and patriotic health campaign, which are not equal to the powers and obligations of the policy issuing agencies, resulting in the phenomenon of ultra virus issuance and scheduling failure in the process of policy implementation. 

**Figure 9:**
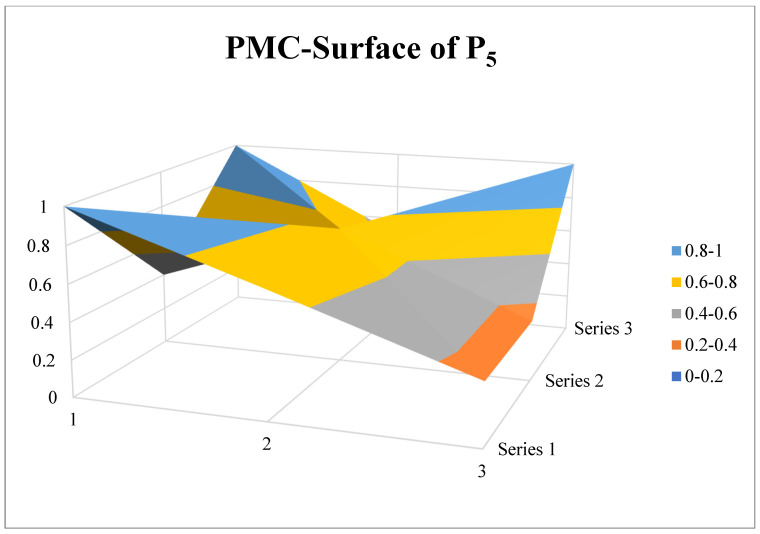
PMC-Surface of P_5_.

P_5_ is “Regulation on emergency response to public health emergencies” issued in 2011 by the State Council, and is the only policy in China with a high level of legal force that directly addresses emergency response to public health emergencies. The curved surface chart (Figure 9) suggests that X_1_ to X_3_ show a straight-line downward trend, and the scores decrease in turn; in addition, there is an obvious depression between X_7_ and X_9._ P_5_’s X_3_, X_4_, X_6_ and X_8_ do not score well for similar reasons as the other policies. In addition to this, as a comprehensive emergency response policy, P_5_ focuses only on guidance for government departments and the public, neglecting the mobilisation and guidance of enterprises and non-profit organisations. 

**Figure 10:**
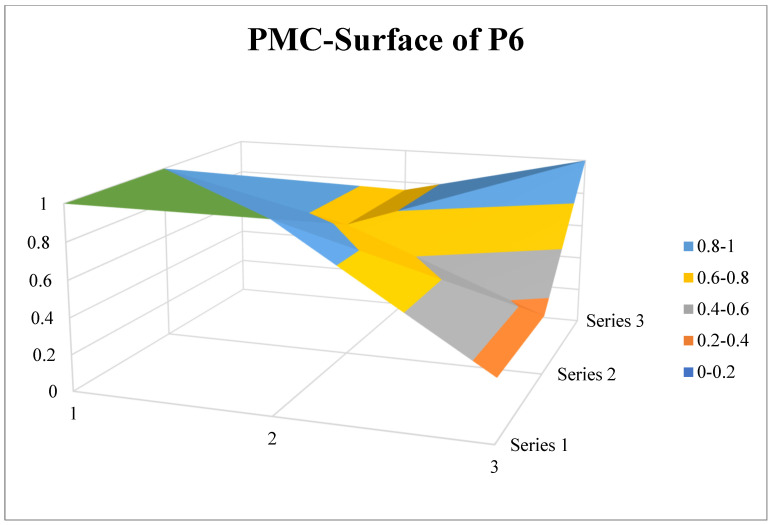
PMC-Surface of P_6_.

P_6_ is the law of the People’s Republic of China on the prevention and control of infectious diseases revised in 2013, and is also the most effective policy text in the field of infectious disease prevention and control. For the prevention and control of infectious diseases, the policy establishes a set of strict standard systems for prevention, reporting, control, medical treatment, supervision and management, and safeguard measures, defines the responsibilities of governments at all levels and provides effective operational guidance for grass-roots departments. Looking at Figure 10, it can be seen that there is a limited range of concavity in the surface plot of P_6,_ excluding the common problem of X_3_ and X_6_, the other level indicators of P_6_ are generally relatively balanced, with only X_7_ scoring below average for the policy mode of action. This is mainly due to the lack of incentive measures for the prevention and control of infectious diseases, excessive reliance on mandatory and service measures, long-length description of legal liability and incentive provisions such as granting incentives and allowances are only briefly covered in Chapter VII safeguards. 

## 5. Conclusions

Public health events that may occur at any time in a risk society pose a great threat to people’s lives and health, and the process of globalization has closely linked the fate of all countries in the world together. The global outbreak and spread of public health problems, represented by the COVID-19 outbreak, has brought a huge challenge to public health security systems [67]. The prevention of and response to public health emergencies in a large, open country with a large population base such as China is of great importance to the defence of global public health.

After the SARS crisis in 2003, China’s ability to prevent and control infectious diseases has improved slightly; subsequently, during public health events such as Influenza A (H1N1), Ebola importation and COVID-19, China introduced a series of policies to safeguard the lives and property of its citizens and maintain a stable social order to counteract the risks; thus, the public health emergency management system has been gradually improved and the government’s ability to prevent and respond to public health emergencies has been enhanced, step by step [68].

In this context, this paper carried out text retrieval, policy sorting, text analysis and quantitative evaluation on 327 central-level PHERP since 1989–2022 which can represent the nation’s will on prevention and emergent control of public health crises. The outcome of the analysis indicates that the design of China’s PHERP is relatively reasonable and systematic. Even though policy content is not composed of numbers, we introduced the PMC-Index into our research, which created some possibilities to quantitatively evaluate and score the policies. In general, it is obvious that PHERP has played an important role in the process of resisting public health risks, comprehensively guaranteed the health of all citizens, maintained the peace of social order and strengthened the public’s trust in the government. However, restricted by the development of social and public health prevention and control scientific knowledge, policy content inevitably lags behind, thus, there is still some weakness in the policy texts.

This paper presents a textual analysis of 327 policies with the help of natural language processing tools. Through the policy content analysis, we found that the main objective of PHERP is to improve China’s ability to prevent, control and deal with public health emergencies and protect the physical and mental health of all citizens. However, the results of high-frequency vocabulary statistics suggest that the current policy still mainly focuses on emergency response after the event, and less attention is paid to the prevention of emergencies; in addition, the attention that is paid to the prevention and control of public health emergencies has been limited to infectious disease prevention. At the same time, the results of the semantic network analysis show that at this stage, China’s public health emergency response policies are mainly formulated and issued by the Ministry of Health, and the issuance of policies relies mostly on the form of notice, which has resulted in a low level of policy effectiveness and integration between policies and a high level of redundancy in policy content.

By introducing PMC-Index, designing an indicator system and constructing PMC-Surface, we are able to evaluate the selected representative ten policies. The scores of ten policies are all above 4, which means all policies are ranked as acceptable and higher level, with two policies rated Optimal, six policies rated Good, and the remaining two all rated Acceptable. However, the unevenness in PMC-Surface indicates that the scores of main indicators for various policies are still unbalanced. Besides the total score of each policy, there is also a relatively large gap between the average score of each main indicator.

In terms of policies, their scores, from highest to lowest, were P_10_, P_3_, P_4_, P_6_, P_2_, P_1_, P_5_, P_9_, P_7_ and P_8._ The highest average score of the main indicators is policy nature X_1_, and the lowest is policy time X_3_ and policy release agency X_6_. Furthermore, it is interesting to note that the ten policies scored the same in X_3_ and X_6_, including P_10_, which ranked first, and X_3_ and X_6_ are the only two indicators that cannot score full marks for P_10_. This makes it clear that X_3_ and X_6_ are the common weaknesses of all ten policies.

These ten policies are all effective policies with a long-time limit, which to some extent reduces the possibility of a policy being changed capriciously. However, the long-term implementation of the policy is also faced with the problem of fatigue in the implementation process, so it is particularly important to set assessment objectives for the policy in stages in the policy text. However, throughout the ten policies, there is no task unbundling and implementation assessment for the time-bound completion of the policy objectives, and there is a lack of medium and short-term policy plans, so none of the policies score well in terms of timeliness. In addition, there is a lack of inter-subject coalition in the publication of policies, with all ten being published by a single institution, a result that is highly consistent with the outcome of the semantic network analysis. While we advocate professional bodies to give full play to their expertise and formulate professional response policies, the public can be overwhelmed by the sheer number of response policies. On the other hand, the secret of P_10_, which scored the highest, is that it incorporates all the response processes in its content, including medical treatment, prevention, recovery and normalisation of prevention and control of public health incidents. For the public, reading and understanding one policy provides a basic understanding of the full spectrum of emergency response. In addition, from the scores of the first level indicators of the lower ranking policies, we can easily see that they have a poor grasp of the full range of policy elements.

Based on the results of the above discussion, from the perspective of the main indicator system, this paper makes the following recommendations for the development of future PHERP.

(1) At present, the number of preventive policies is obviously less than the number of emergency response policies, but effective measures can prevent and reduce the harm caused by public health emergencies and, to a certain extent, can guide the public to develop good health habits in life. Therefore, in the future, relevant policies should be issued for the prevention of public health emergencies.
(2) Use the three major policy instruments of “supply–demand–environment” in an integrated manner, strengthen the use of demand-based policy tools, further release China’s institutional advantages in the new situation of risk society, strengthen public–private collaboration and involve businesses, non-profit organizations and others in crisis prevention and response to take advantage of the synergy of multiple actors in governance.
(3) Policy texts should be published with clear policy timeliness. On the one hand, it can restrict the government’s arbitrary change of policies; on the other hand, for policies with a long effective time, it is necessary to add a phased policy target completion assessment scheme to the policy text, which can test the effectiveness of policy implementation and urge the policy implementation department to act in accordance with the policy.
(4) The policy text lacking the unity of government departments tends to result in both a large number of policies and a tendency to overreach on policy making. At the same time, China does not yet have a supreme law that directly addresses the response to public health emergencies. For PHERP, it is important to be able to provide the policy audience with a clear and scientific understanding of their rights and obligations. Therefore, the resources of the publishing bodies should be consolidated as far as possible to avoid the government speaking at cross-purposes, while a policy text with a high legal force should be issued as soon as possible.
(5) Under the guidance of the concept of multi-governance, coordinated prevention and disposal of public health emergencies, the policy text needs to change the mind-set of relying only on traditional mandatory administrative methods to promote policy implementation, shift from crisis management to crisis governance, pay more attention to promoting policy implementation through service-oriented and incentive measures and turn government forced promotion into spontaneous participation.
(6) The current policy target groups are set primarily as government departments and all citizens, with less guidance and arrangements for enterprises and non-profit organizations. However, under the influence of the concept of national pluralistic and collaborative governance, the response to public health events should also attach importance to the reasonable borrowing of non-governmental forces and the expression of their rights and interests, for example, volunteer organizations and various industry associations in emergency response to public health emergencies. The rules of public–private cooperation and their respective rights and obligations should be reflected in the future higher level legal texts dealing with public health emergencies.

## 6. Limitations

Although this paper introduces natural language processing tools and the PMC-Index model into the PHERP domain and analyses existing policies through a combination of qualitative and quantitative method. To some extent, the method is innovative and provides some suggestions for policy improvement from the perspective of primary indicators. However, there are still some limitations of this paper. First, the policy texts are all national-level PHERP and lack attention to local level policies. Secondly, due to the length of the article, it is not possible to calculate the index of all policies and to show the advantages and disadvantages of all policies through the PMC-Surface; Thirdly, the policy indicator system is constructed on the basis of existing research literature and textual analysis and may still have certain gaps. We will continue to focus on public health emergency response policies in the future and continue to improve our evaluation indicator system to provide more scientific, rational and comprehensive suggestions for public health emergency response policy making and to defend the health of all citizens of the planet.

## Figures and Tables

**Table 1:** Main and sub indicators.

Main-Indicators	Sub-Indicators	Source
X_1_ Policy Nature	X_1:1_ Forecast; X_1:2_ Supervise; X_1:3_ Guide; X_1:4_ Support; X_1:5_ Describe; X_1:6_ Proposal	[61,62]
X_2_ Policy Instrument	X_2:1_ Supply-based; X_2:2_ Demand-based; X_2:3_ Environment construction-based	[13,42,63]
X_3_ Policy Time	X_3:1_ Long-term; X_3:2_ Mid-term; X_3:3_ Short-term	[64]
X_4_ Policy Field	X_4:1_ Economics; X_4:2_ Politics; X_4:3_ Education; X_4:4_ Social services; X_4:5_ Social environment	[53]
X_5_ Policy Function	X_5:1_ Medical treatment; X_5:2_ Science popularization and education; X_5:3_ Prevention in advance; X_5:4_ Post recovery and reconstruction; X_5:5_ Permanent prevention and control; X_5:6_ Stabilising social order; X_5:7_ Emergency system construction	[64]; By authors based on the results from content analysis
X_6_ Policy Release Agency	X_6:1_ National People’s Congress; X_6:2_ State Council and General Office of State Council; X_6:3_ National Ministries	[62]
X_7_ Policy Action Mode	X_7:1_ Mandatory; X_7:2_ Service-oriented; X_7:3_ Motivational	[64]
X_8_ Policy Target Groups	X_8:1_ Government departments; X_8:2_ Enterprises; X_8:3_ Non-profit organisations; X_8:4_ Public	[64]
X_9_ Policy Evaluation	X_9:1_ Clear objectives; X_9:2_ Well established; X_9:3_ Reasonable scheme; X_9:4_ Detailed planning; X_9:5_ Clear responsibility and authority	[61,62]
X_10_ Policy Disclosure	No sub-indicators	[53]

**Table 2:** Selected policy texts.

Number	Title	Release Agency	Release Date
P_1_	Measures for the Implementation of the Law of the People’s Republic of China on the Prevention and Treatment of Infectious Diseases	State Council	6 December 1991
P_2_	Notice on the issuance of the outline of the tenth five-year plan for national preventive control	General Office of the Ministry of health	7 September 2001
P_3_	National Emergency Response Plan for Public Health Emergencies	State Council	26 February 2006
P_4_	Notice on the issuance of the National Health Emergency Plan for Natural Disasters (for trial implementation)	Ministry of Health	27 April 2009
P_5_	Emergency Regulations for Public Health Emergencies	State Council	8 January 2011
P_6_	Law of the People’s Republic of China on the Prevention and Treatment of Infectious Diseases	Standing Committee of the National People’s Congress	29 June 2013
P_7_	Notice on the issuance of the Public Health Prevention, Control and Treatment Capacity Building Program	National Development and Reform Commission, National Health Commission, and State Administration of Traditional Chinese Medicine	9 May 2020
P_8_	Notice on the Issuance of the Regulations on Food Safety Risk Monitoring	National Health Commission	4 November 2021
P_9_	Notice on printing and distributing the guidelines for the construction and management of national TCM emergency medical teams (for Trial Implementation)	Office of the State Administration of traditional Chinese Medicine	11 June 2021
P_10_	National health plan of the 14th five-year plan	General Office of the State Council	27 April 2022

**Table 3:** Results of high frequency vocabulary statistics.

Number	Word	Frequency	Number	Word	Frequency	Number	Word	Frequency
1	Hygiene	9169	2	work	9122	3	Emergency	6430
4	Management	4388	5	Disease	4196	6	Strengthen	4063
7	Epidemic	3704	8	Information	3683	9	Monitor	3402
10	Public Health	3019	11	Event	3010	12	Country	2950
13	Control	2680	14	Organize	2553	15	Cases of Disease	2547
16	Prevention and Control	2230	17	Unit	2204	18	Testing	2197
19	Timely	2111	20	Health Care	1896	21	Prevention and Treatment	1887
22	Training	1698	23	Hospital	1678	24	Patient	1600
25	Investigation	1553	26	Evaluation	1532	27	Control Centre	1507
28	Region	1465	29	Specialty	1439	30	Infection	1412
31	Health	5541	32	Agency	4669	33	Prevention	4006
34	Infectious Diseases	3718	35	Service	3361	36	Report	3320
37	Burst	2936	38	Technology	2711	39	Personnel	2433
40	Medical care	2385	41	Administrative Department	2146	42	Capability	2127
43	Data	1868	44	Indicator	1750	45	Provincial Level	1577
46	Education	1574	47	Medical Institution	1483	48	Standard	1478
49	Treatment	1408	50	Norm	1401			

**Table 4:** Multi-input–output table.

X_1_						X_2_		
X_1:1_	X_1:2_	X_1:3_	X_1:4_	X_1:5_	X_1:6_	X_2:1_	X_2:2_	X_2:3_
X_3_				X_4_				
X_3:1_	X_3:2_	X_3:3_		X_4:1_	X_4:2_	X_4:3_	X_4:4_	X_4:5_
X_5_						X_6_		
X_5:1_	X_5:2_	X_5:3_	X_5:4_	X_5:5_	X_5:6_	X_6:1_	X_6:2_	X_6:3_
X_7_			X_8_					
X_7:1_	X_7:2_	X_7:3_	X_8:1_	X_8:2_	X_8:3_	X_8:4_		
X_9_								
X_9:1_	X_9:2_	X_9:3_	X_9:4_	X_9:5_				

**Table 5:** Results of PMC-Index.

Policy	X_1_	X_2_	X_3_	X_4_	X_5_	X_6_	X_7_	X_8_	X_9_	X_10_
P_1_	1	1	0.33	0.6	0.75	0.33	0.67	0.5	1	1
P_2_	1	1	0.33	0.8	0.75	0.33	0.67	1	0.8	1
P_3_	1	1	0.33	0.6	1	0.33	1	0.75	1	1
P_4_	1	1	0.33	0.8	0.88	0.33	1	0.5	1	1
P_5_	1	0.67	0.33	0.4	0.75	0.33	1	0.5	1	1
P_6_	1	1	0.33	1	0.75	0.33	0.67	0.75	1	1
P_7_	1	0.67	0.33	0.6	0.5	0.33	0.33	0.25	0.8	1
P_8_	1	0.67	0.33	0.2	0.38	0.33	0.33	0.75	0.8	1
P_9_	1	0.67	0.33	0.8	0.5	0.33	0.67	0.25	1	1
P_10_	1	1	0.33	1	1	0.33	1	1	1	1

**Table 6:** Rank of policy.

Number	PMC-Index	Evaluation Level	Rank
P_1_	7.18	Good	6
P_2_	7.68	Good	5
P_3_	8.01	Optimal	2
P_4_	7.84	Good	3
P_5_	6.98	Good	7
P_6_	7.83	Good	4
P_7_	5.81	Acceptable	9
P_8_	5.79	Acceptable	10
P_9_	6.55	Good	8
P_10_	8.66	Optimal	1

## References

[1] WHO (2005). International Health Regulations.

[2] Heinrich H.W., Petersen D.C., Ross N.R. (1980). Industrial Accident Prevention.

[3] Marshall P., Hirmas A., Singer M. (2018). Heinrich’s pyramid and occupational safety: A statistical validation methodology. Saf. Sci..

[4] State Council of China (2003). Emergency Regulations for Public Health Emergencies.

[5] An L., Yu C., Lin X., Du T., Zhou L., Li G. (2018). Topical Evolution Patterns and Temporal Trends of Microblogs on Public Health Emergencies. Online Inf. Rev..

[6] Kriger D. (2021). What is risk? Four approaches to the embodiment of health risk in public health. Health Risk Soc..

[7] Beck U., Zhang W., He B. (2018). Risk Society.

[8] Wang Y., Cheng W. (2022). From Beck to Giddens: Epistemological differences in risk society theory. Soc. Sci. Res..

[9] Beck U., Wilms J., Lu G. (2001). Freedom and Capitalism.

[10] Giddens A. (1990). The consequences of Modernity.

[11] Campbell A.L. (2012). Policy makes mass politics. Annu. Rev. Political Sci..

[12] Yin Y. (2019). Public Policy evaluation: The coupling of rationalism and constructivism. Chin. Public Adm..

[13] Zhang J., Zhao R., Guo H., Wei M., Su Y. (2022). Quantitative analysis of policy tools for coping with legal texts for public health emergencies in China since SARS event. Chin. Health Serv. Manag..

[14] Yao M., Gui Q. (2020). Study on local public health legislation in China—An empirical analysis based on 32 local laws and regulations. Chin. Health Serv. Manag..

[15] Wu K., Wu C. (2022). Research on differences in policy response speed under the background of major public health emergencies—Event history analysis based on policies for resumption of work and production in 283 cities. J. Beijing Univ. Technol..

[16] Lyu H., Wang W., Yan Z., Hou L. (2021). Competitive neutrality and non-state-owned enterprises’ access to debt financing: A quasi-natural experiment during the covid-19 pandemic. J. Financ. Res..

[17] Xu C., Duan G. (2021). Research on policies and regulations on the application of citizen location information in the prevention and control of public health emergencies. Soc. Sci. Guangxi.

[18] Zhang Z. (2022). National public health governance system transformation and adaptive crisis-and the value of policy in the early transmission phase of an epidemic. Tianjin Soc. Sci..

[19] Liu W., Guo J., Shi D. (2021). How to evaluate public policy scientifically? The counterfactual framework of policy evaluation studies and matching methods. J. Public Adm..

[20] Wen H., Zheng H. (2022). On differences about public sentiment feedback of public policies making in major public health emergencies—An investigation about the policy of “delay of school opening” based on social media data. J. Beijing Univ. Technol..

[21] Li F., Ma L., Li S. (2018). An evidence-based approach to public policy evaluation—Experimental design and casual inference. J. Chin. Acad. Gov..

[22] Sanderson I. (2010). Evaluation, policy learning and evidence-based policy making. Public Adm..

[23] Gibson P.J., Theadore F., Jellison J.B. (2012). The Common Ground Preparedness Framework: A Comprehensive Description of Public Health Emergency Preparedness. Am. J. Public Health.

[24] Hodge J.G., Gostin L.O., Vernick J.S. (2007). The Pandemic and All-hazards Preparedness Act-Improve Public Health Emergency response. J. Am. Med. Assoc..

[25] Bai Y., Tang Y. (2020). Comparative research on China’s fiscal and tax policies in response to public health emergencies. Econ. Theory Bus. Manag..

[26] Fan Y., Yang S., Jia P. (2020). Preferential Tax Policies: An Invisible Hand behind Preparedness for Public Health Emergencies. Int. J. Health Policy Manag..

[27] Dai J. (2021). The combination of employment policy instruments for governance of a major public health crisis: A text analysis based on Provincial-Level policy. China Public Adm. Rev..

[28] Ma W., He X., Chen X. (2022). The impact of supportive policies and their combination on micro, small, and medium-sized enterprises’ resumption of work and production: A PSM analysis in the context of COVID-19. Soft Sci..

[29] Peng W. (2022). Criminal policy model under major public health events. Glob. Law Rev..

[30] Zhao B., Yuan B. (2020). On criminal policy for prevention and control of major public health events in China: Focus on criminal policy of China’s novel Coronavirus epidemic prevention and control. Jianghai Acad. J..

[31] Liu J., An L. (2022). Policy gaps and bridging strategies for personal information protection in the context of public health emergencies. Libr. Trib..

[32] Myers N., Bearss A. (2018). Mandating public health emergency preparedness: Analysis of the CMS Rule. Risk Hazards Crisis Public Policy.

[33] Li Y., Wang X. (2022). Experience and enlightenment of European and American personal information protection laws and policies in public health emergencies. Inf. Doc. Serv..

[34] Zhang Y., Zhou Y. (2017). Policy instrument mining and quantitative evaluation of new energy vehicles subsidies. China Popul. Resour. Environ..

[35] Sun M. (2014). Policies change related to public health emergency disposal in China: From 2003 to 2013. Chin. J. Health Policy.

[36] Hu G., Rao K., Sun Z., Sun Z. (2007). An investigation into local government plans for public health emergencies in China. Health Policy Plan..

[37] Chen W. (2022). Exploration of the governance model of public health emergencies: From the perspective of risk society. J. Beijing Univ. Aeronaut. Astronaut..

[38] Fu Y. (2015). Quantitative analysis of public policy: Reason and value of the research paradigm transformation. China Public Adm..

[39] Cai L., Hu X. (2022). Research on the non-equilibrium and optimization of China’s public health emergency management policy based on text analysis. J. Shanghai Adm. Inst..

[40] Pei J., Zhou X., Zhang R., Yu X. (2021). Analysis and research on the government informaion reporting system of public health emergencies based on policy texts. Inf. Doc. Serv..

[41] Ma X., Zhang X., Qin C. (2020). Research on the sudden public health incidents response policies based on policy tools: Taking COVID-19 as an example. Inf. Stud. Theory Appl..

[42] Zhang R., Zhou X., Pei J., Yu X. (2021). Quantitative analysis of policy tools for emergency information management of public health emergencies in China. Inf. Doc. Serv..

[43] Ignatow G. (2016). Theoretical Foundations for Digital Text Analysis. J. Theory Soc. Behav..

[44] Aiden E., Michel J. (2013). Uncharted: Big Data as a Lens on Human Culture.

[45] Zhang C., Guan J. (2020). Based on the evaluation of a policy system by the content analysis on policy texts: Evidence from the innovation and entrepreneurial policy system in china. Manag. Rev..

[46] Jin X., Min J., Ju J. (2022). Evolution of China’s Equity Pledge Policy. Soc. Sci..

[47] Estrin S., Mickiewicz T., Stephan U. (2016). Human Capital in Social and Commercial Entrepreneurship. J. Bus. Ventur..

[48] Guan J., Liu N. (2016). Exploitative and Exploratory Innovations in Knowledge Network and Collaboration Network: A Patent Analysis in the Technological Field of Nano-Energy. Res. Policy.

[49] Lina M., Rojas B. (2016). Deep Learning for Sentiment Analysis. Lang. Linguist. Compass.

[50] Li D., Li L., Li D. (2021). The Public Opinion Effect of the Introduction of the Three-Child Policy and Implications—Network big data analysis based on NLP. China Youth Study.

[51] Kastrati Z., Dalipi F., Imran A. (2021). Sentiment Analysis of Students’ Feedback with NLP and Deep Learning: A Systematic Mapping Study. Appl. Sci..

[52] Chen Q., Zhang Y. (2022). Evaluation of Carbon reduction policies for animal husbandry—An analysis based on 452 policies. J. Huazhong Agric. Univ..

[53] Estrada M.R. (2011). Policy Modeling: Definition, Classification and Evaluation. J. Policy Model..

[54] Lu C., Wang B., Chen T., Yang J. (2022). A Document Analysis of Peak Carbon Emissions and Carbon Neutrality Policies Based on a PMC Index Model in China. Int. J. Environ. Res. Public Health.

[55] Li Z., Guo X. (2022). Quantitative evaluation of China’s disaster relief policies: A PMC index model approach. Int. J. Disaster Risk Reduct..

[56] Li Y., He R., Liu J., Li C., Xiong J. (2022). Quantitative Evaluation of China’s Pork Industry Policy: A PMC Index Model Approach. Agriculture.

[57] Li G., Lan S., Jiang X. (2007). Methods for Content Analysis of Public Policy: Theory and Application.

[58] Doerfel M.L., Barnett G.A. (2010). A semantic network analysis of the international communication association. Hum. Commun. Res..

[59] Dai S., Zhang W., Zong J., Wang Y., Wang G. (2021). How effective is the green development policy of chine’s Yangtze River economic belt? A quantitative evaluation based on the PMC-Index model. Int. J. Environ. Res. Public Health.

[60] Estrada M.R., Yap S.F., Nagaraj S. (2007). Beyond the Ceteris Paribus Assumption: Modeling Demand and Supply Assuming Omnia Mobilis.

[61] Zhang Y., Geng Z. (2015). The Quantitative Evaluation of Regional Science and Technology Innovation Policy: Based on The Index of PMC Model. Sci. Technol. Manag. Res..

[62] Xiao J. (2022). The Quantitative Evaluation of Women’s Employment Security Policies at The Central Government Level. J. Shandong Womens Univ..

[63] Rothwell R., Zegveld W. (1985). Reindusdalization and Technology.

[64] Liu G., Han W., Chen A. (2021). Quantitative study of public health emergency response policies based on a three-dimensional analysis framework—Taking the COVID-19 as an example. J. Mod. Inf..

[65] Yu Y., Gao K. (2022). The embedding of sports social organization in rural governance based on the collaborative governance model of multiple subjects. Math. Probl. Eng..

[66] Shiroyama H., Yarime M., Matsuo M., Schroeder H., Scholz R., Ulrich A.E. (2012). Governance for sustainability: Knowledge integration and multi-actor dimensions in risk management. Sustain. Sci..

[67] Peter S. (2020). The New Epidemic Strikes the Death Knell of Globalization.

[68] Zhang J., Li C., Gu Y., Zeng X., Fan L., Shi L. (2020). Examination on management policies for public health emergency based on punctuated equilibrium theory. Med. Philos..

